# The effects of exercise programs on cognition, activities of daily living, and neuropsychiatric symptoms in community-dwelling people with dementia—a systematic review

**DOI:** 10.1186/s13195-022-01040-5

**Published:** 2022-07-22

**Authors:** Kathrin Steichele, Anne Keefer, Nikolas Dietzel, Elmar Graessel, Hans-Ulrich Prokosch, Peter L. Kolominsky-Rabas

**Affiliations:** 1 Friedrich-Alexander-Universität Erlangen-Nürnberg, Interdisciplinary Center for Health Technology Assessment and Public Health (IZPH), Erlangen, Germany; 2 Department of Psychiatry and Psychotherapy, Center for Health Services Research in Medicine, University Hospital Erlangen, Erlangen, Germany; 3 Friedrich-Alexander-Universitat Erlangen-Nurnberg, Department of Medical Informatics, Biometrics and Epidemiology, Erlangen, Germany

**Keywords:** Non-pharmacological treatment, Physical activity, Cognitive function, BPSD, ADLs

## Abstract

**Background:**

The prevalence of dementia is expected to increase dramatically. Due to a lack of pharmacological treatment options for people with dementia, non-pharmacological treatments such as exercise programs have been recommended to improve cognition, activities of daily living, and neuropsychiatric symptoms. However, inconsistent results have been reported across different trials, mainly because of the high heterogeneity of exercise modalities. Thus, this systematic review aims to answer the questions whether exercise programs improve cognition, activities of daily living as well as neuropsychiatric symptoms in community-dwelling people with dementia.

**Methods:**

Eight databases were searched for articles published between 2016 and 2021 (ALOIS, CENTRAL, CINAHL, Embase, MEDLINE, PsycINFO, PubMed, Web of Science). Randomized controlled trials evaluating the effects of any type of physical activity on cognition, activities of daily living, or neuropsychiatric symptoms in community-dwelling people with a formal diagnosis of dementia were included in this systematic review. Two authors independently assessed eligibility and quality of the studies. The methodology was in line with the Preferred Reporting Items for Systematic Reviews and Meta-Analysis guidelines.

**Results:**

Eight publications covering seven trials were included in this review with the majority investigating either a combination of strength and aerobic exercise or aerobic exercise alone. This review revealed that there is no clear evidence for the beneficial effects of exercise on cognition. None of the included trials found an impact on activities of daily living. Although different randomized controlled trials reported inconsistent results, one trial indicated that especially aerobic exercise may improve neuropsychiatric symptoms.

**Conclusion:**

Our systematic review did not confirm the impact of exercise on cognition and activities of daily living in community-dwelling people with dementia. The results suggested that aerobic exercise might be effective to reduce neuropsychiatric symptoms. Well-designed trials including only community-dwelling people with a formal diagnosis of dementia, large samples, long-term follow-ups, and detailed description of adherence to the intervention are needed to improve the scientific evidence on the best type of exercise modality.

**Trial registration:**

PROSPERO, CRD42021246598.

**Supplementary Information:**

The online version contains supplementary material available at 10.1186/s13195-022-01040-5.

## Introduction

Improvements in health care in the past decades have contributed to an increase in life expectancy. Although dementia is not an inevitable part of normal aging, incidence increases with age. Currently, over 55 million people worldwide live with dementia, and prevalence of dementia is expected to increase dramatically as the population ages [1].

Due to the limited availability of pharmacological treatment, non-pharmacological interventions have been recommended as first-line approaches for over a decade to improve cognition, activities of daily living (ADLs), and neuropsychiatric symptoms (NPS) in people with dementia (pwd) [2, 3]. Among these, exercise has been recommended as an effective treatment for slowing down cognitive decline in pwd [4–7]. Furthermore, recent research partially shows physical activity to be a promising method to reduce NPS and improve ADLs [4, 5, 8]. However, these findings are not consistent, and conflicting results have been reported across different trials [9–11]. Although this is a field of high interest and many trials have been conducted, recent evidence seems controversial, as various systematic reviews reported conflicting results [6, 12–14]. As people with mild cognitive impairment (MCI), pwd living in a long-term care facility, and community-dwelling pwd have different needs and capabilities, it is necessary to distinguish between these groups, which has not been done in previous reviews. Thus, this systematic review aims to give a broad overview of the effects of exercise programs on cognition, ADLs, and NPS in community-dwelling people with a formal diagnosis of dementia. Moreover, frequency, intensity, duration, and setting of the interventions have been hardly discussed in previous reviews. Therefore, we aim to analyze how training modalities (type of training, frequency, duration, intensity, setting) influence the effects of exercise.

## Methods

This systematic review was conducted and reported following the guidelines of Preferred Reporting Items for Systematic Reviews and Meta-Analysis (PRISMA) [15] and was registered at the national prospective register of systematic reviews (PROSPERO registration number: CRD42021246598).

### Data sources and searches

PubMed, MEDLINE, Embase, ALOIS, Web of Science, PsychINFO, CINAHL, and CENTRAL were systematically searched using different terms for exercise and dementia. Full search strategy can be found in Additional file 2. Whenever possible, searches with additional filters such as randomized controlled trials as article type, English or German as language and publication date from 2016 until 2021, were conducted. Since a Cochrane Review on this topic has been conducted in 2015, we aim to focus on trials published after 2015.

### Study selection

Randomized controlled trials (RCTs) over any length of time with the aim of improving cognition, ADLs, or NPS in pwd were eligible for this systematic review. As an intervention, we included RCTs providing any combination of resistance, endurance, or balance training. Multidomain interventions in which isolated effects of exercise cannot be measured (e.g., combination with cognitive training) had to be excluded. For control groups, usual care or social activities were included, while following regular exercise was used as exclusion criteria. Furthermore, we eliminated trials in which people with MCI or subjective cognitive impairment (SCI) or institutionalized pwd were involved. We considered people living in the community or assisted-living facilities at the time of intervention which is why acute hospitalized pwd or people living in long-term care facilities were excluded. Since most of the pwd living at home with community-dwelling people having different capabilities than institutionalized pwd, we excluded institutionalized pwd, where the disease is often more advanced [16, 17]. We had no restrictions regarding the type of dementia, as long as they had a formal medical diagnosis.

After merging search results and discarding duplicates, title, abstracts, and full texts were independently screened for inclusion by the first two authors (K.S. and A.K). In cases of disagreements, the last author was consulted for the final decision (P.K.-R.).

### Data extraction and risk of bias assessment

Information from the articles was extracted by the first author, with the second author checking the collected data, which included study setting, publication year, country, way of recruitment, funding, inclusion and exclusion criteria, sample size, and drop-out rates.

Extracted data also covered participants’ baseline characteristics such as gender, age, diagnostic criteria, and diagnosis as well as Mini-Mental Status Examination (MMSE) score at baseline when available. Furthermore, a detailed description of the exercise modalities (e.g., type, frequency, duration, actual and planned intensity) and outcome data of the first follow-up was gathered. If a study combined multiple types of exercise, it was regarded as a multimodal intervention. Between-group differences in the mean changes from baseline to follow-up in domains of cognition, ADLs, and NPS were reported.

The quality of included studies was assessed by the first and the third authors using the “Revised Cochrane Risk-of-Bias Tool for Randomized Trials (RoB2)” [18].

## Results

### Included studies

**Fig. 1:**
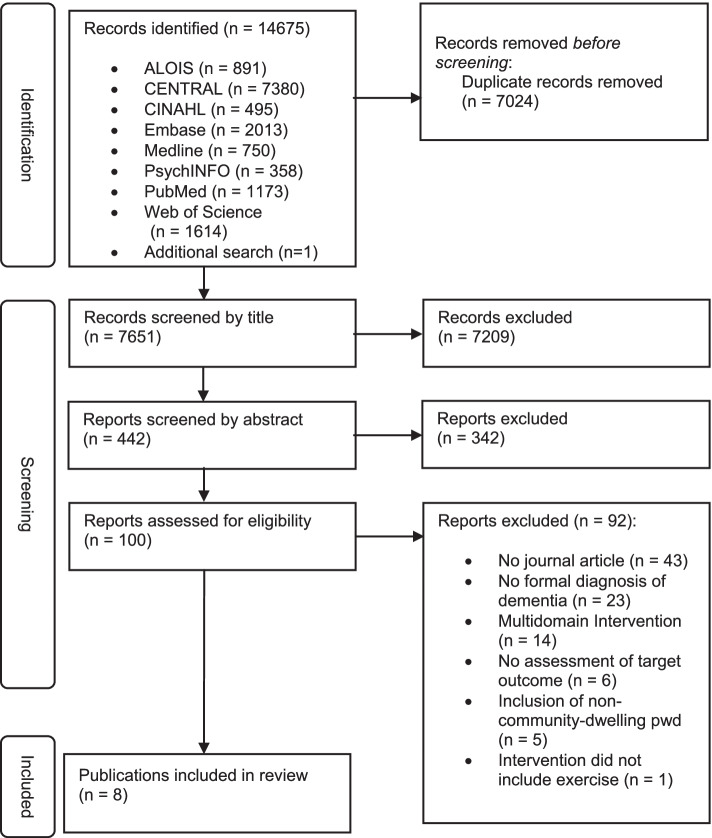
Flow diagram of the literature search and trials selection process (based on PRISMA)

In total, 14,675 studies were identified through the search. After removing duplicates, 7651 records were screened of which 7551 were excluded as they covered irrelevant topics (Fig. 1). Of the remaining 100 articles, 92 studies were further excluded mainly because of: (1) other publication formats such as conference papers, (2) participants without a formal diagnosis [8, 19–22], (3) a combination of different interventions [23–28], (4) no assessment of target outcome [29–31], (5) or institutionalized pwd [32–36].

Consequently, eight publications of seven trials with a total of 1135 participants were included in this review. Detailed study characteristics are summarized in Table 1. The mean age ranged from 70.5 ± 7.4 [37] to 84.3 ± 7.7 years [38] and participants baseline MMSE varied between 14.7 ± 5.65 [38] and 23.94 ± 3.6 [37]. Different types of exercise were conducted, with the majority of included studies investigating either aerobic training [37, 39, 40] or a combination of aerobic, strength, and balance training (multimodal training) [41–43]. With the exception of Park et al. [38], all trials examined the effects of exercise on cognition. Effects on ADLs were investigated in two studies [37, 42] and NPS in four studies [37, 38, 42, 44]. Adherence to the protocol ranged from 65.63% [42] to 87% [26].

**Table 1:** Study characteristics of included studies

Study	Country	Sample size		Age [years]		Gender [% female]		MMSE (mean ± SD)		Diagnosis criteria	Type of training		Trial duration [weeks]	Relevant outcomes	Risk of bias
		IG	CG	IG	CG	IG	CG	IG	CG		IG	CG			
De Oliveira et al. [41]	Brazil	13	14	77.54 ± 8.05	81.22 ± 8.88	78.6	38.5	20.9 ± 4.34	20.66 ± 5.19	DSM-IV	Multimodal training	Usual care	12	Cognition	High concerns
Hoffmann et al. [37]	Denmark	107	93	69.8 ± 7.4	71.3 ± 7.3	47.7	38.7	23.8 ± 3.4	24.1 ± 3.8	NINDS-ADRDA	Aerobic training	Usual care	16	Cognition ADLs NPS	Some concerns
Karssemeijer et al. [39]	The Netherlands	39^a^	38	80.9 ± 6.1	79.8 ± 6.5	44.7	46.2	22.5 ± 3.1	21.9 ± 3.1	DSM-IV	Aerobic training	Stretching	12	Cognition	Some concerns
Lamb et al. [42]	UK	329	165	76.9 ± 7.7	78.1 ± 7.7	40	37	22.1 ± 4.6	22.1 ± 4.6	DSM-IV	Multimodal training	Usual care	16	Cognition ADLs NPS	Some concerns
Öhman et al. [43, 44]	Finland	70	70	77.5 ± 5.4	78.1 ± 5.3	42.9	37.1	17.8 ± 6.6	17.7 ± 6.2	NINDS-ADRDA	Multimodal training	Usual care	52	Cognition NPS	High concerns
		70		78.3 ± 5.1		35.7		18.5 ± 6.3							
Park et al. [38]	USA	11	10	84.3 ± 7.7		41.9		14.7 ± 5.65		NI	Strength training	Music intervention	12	NPS	High concerns
		10									Chair yoga				
Yu et al. [40]	USA	64	32	77.4 ± 6.6	77.5 ± 7.1	44	47	21 ± 3.5	22.2 ± 2.7	NINDS-ADRDA	Aerobic training	Stretching	24	Cognition	High concerns

*ADLs* Activities of daily living, *NPS* Behavioral and psychological symptoms of dementia, *CG* Control group, *SM-IV* Diagnostic and Statistical Manual of Mental Disorders—fourth edition, *IG* Intervention group, *MMSE* Mini-Mental State Examination, *NINDS-ADRDA* National Institute of Neurological and Communicative Diseases and Stroke/Alzheimer’s Disease and Related Disorders Association

^a ^RCT with 2 intervention groups, but one did not meet inclusion criteria of this review

### Effects of exercise on cognition

Of the seven included trials, six examined the effects of exercise on cognition in pwd, with five trials covering global cognition, measured by the Mini-Mental State Examination (MMSE) [37, 41, 43] or Alzheimer’s Disease Assessment Scale (ADAS-Cog) [40, 42]. Although Yu et al. [40] concluded that aerobic exercise is effective to reduce global cognitive decline, none of the studies demonstrated a significant superiority of the intervention group performing either aerobic [37, 39, 40] or multimodal training [41–43] compared to the control group receiving usual care.

Assessment tools for cognitive subdomains varied between trials. Executive function was assessed in four studies [39–41, 43], either using the Clock Drawing test or composite scores of multiple tests, with only one trial finding mild positive effects after 52 weeks of multimodal training [43] (Table 2). None of the trials reported an impact on the domains verbal fluency and language [37, 40, 41, 43] as well as attention and processing speed [37, 40, 41]. While the effects of exercise through either aerobic [39] or multimodal training [37] on psychomotor speed were analyzed in two trials, only Karssemeijer et al. [39] demonstrated that twelve weeks of aerobic training leads to improvements in psychomotor speed.

**Table 2:** Included studies evaluating the effect of exercise programs on cognition

Study	Type of training	Trial duration [weeks]	Frequency [sessions/week]	Intensity	Session duration [min]	Setting	Adherence	Assessment tool	Domain	Outcome Baseline (mean ± SD)			Outcome Follow-up (mean ± SD)		*p* value	Conclusion
										IG		CG	IG	CG		
De Oliveira Silva et al. [41]	Multi-modal training	12	2	70–80% VO_2_max	60	Supervised	87%	MMSE	Global cognition	20.58 ± 4.91		21.43 ± 4.18	20.31 ± 4.68	19.5 ± 5.37	.11	No effect
								CDT	Executive function	1.5		1	1	1	.54	
								VF	Verbal fluency	12		9	10	10	.16	
								ST3	Selective attention, inhibitory control, and processing speed	42		41.53	43.51	40.08	.93	
Hoffmann et al. [37]	Aerobic training	16	3	70–80% HRmax	60	Supervised groups of 2–5	76%	SDMT	Mental speed and attention	27.1 ± 14.7		25.4 ± 14.3	26.2 ± 15.6	24.1 ± 14.9	.179	No effect
								ADAS-Cog verbal memory test	Verbal memory (immediate recall)	11.2 ± 4.5		11.2 ± 4.5	11.2 ± 4.6	11.4 ± 4.4	.813	
								VF	Verbal fluency	23.2 ± 12		24.2 ± 12.4	23.1 ± 11.8	25.6 ± 14.9	.133	
								SCWT	Psychomotor speed	17.6 ± 10.2		18 ± 9.6	17.5 ± 10.4	18.3 ± 9.9	.441	
								MSSE	Global cognition	23.8 ± 3.4		24.1 ± 3.8	23.9 ± 3.4	23.9 ± 3.9	.244	
Karssemeijer et al. [39]	Aerobic training	12	3	50–75% HRR	30–50	Supervised individually	81.1%	*Z*-score (TMT-B short, SCWT, VF, and RS)	Executive function	0.05 ± 0.72		− 0.03 ± 0.8	0.15 ± 0.74	− 0.12 ± 0.87	.338	No effect
								*Z*-score (LLT)	Episodic memory	− 0.08 ± 1.05		− 0.08 ± 0.86	− 0.11 ± 1.15	− 0.34 ± 1.21	.184	
								*Z*-score (WAIS-III DST and WMS-III SS)	Working memory	0.02 ± 0.73		0.15 ± 0.95	0.04 ± 0.80	− 0.12 ± 1.02	.153	
								*Z*-score (TMT-A and SCWT)	Psychomotor speed	0.14 ± 0.73		0.0 ± 0.81	0.32 ± 0.64	− 0.25 ± 1.04	.004^a^	Favors intervention
								*Z*-score (WMS-R LM and HVLT-R)	Episodic memory	NI					.447	No effect
Lamb et al. [42]	Multi-modal training	16	2 (+ 1)^b^	Moderate to hard	60–90	Supervised groups of 6–8 and unsupervised home-based	65.63%	ADAS-Cog	Global cognition	21.4 ± 9.6	21.8 ± 7.7		22.9 ± 11.6	22.4 ± 9.4	.24	No effect
Öhman et al. [43]	Multi-modal training	52	2	NI	60	Supervised home based	72.88%	CDT	Executive function	2.32 ± 2.04	2.45 ± 2.09		NI		.03^a^	Favors intervention
										2.31 ± 2.09					.07	No effect
								VF	Verbal fluency	8.34 ± 4.75	7.89 ± 4.25		NI		.93	
						Supervised groups of 10	72.12%			8.05 ± 4.3						
								MMSE	Global cognition	17.8 ± 6.6	17.7 ± 6.2		NI		.74	
										18.5 ± 6.3						
Yu et al. [40]	Aerobic training	24	3	50–75% HRR	40–60	Supervised groups up to 3	> 70%	ADAS-Cog	Global cognition	19.3 ± 7.4	17.8 ± 6.5		NI		.386	No effect
								*Z*-score (WMS-R LM and HVLT-R)	Episodic memory	NI					.447	
								*Z*-score (TMT-B, EXIT-25, and CDT)	Executive function	NI					.849	
								WAIS-R DST	Attention	NI					.539	
								*Z*-score (WAIS-III DST and TMT A)	Processing speed	NI					.778	
								*Z*-score (COWAT, VF, and BNT)	Language	NI					.925	

*ADAS-Cog *Alzheimer’s Disease Assessment Scale – Cognitive Subscale, *BNT* Boston Naming Test, *CDT* Clock-Drawing Test, *CG* Control group, *COWAT* Controlled Oral Word Association Test, *HRmax* Maximal heart rate, *EXIT25* Executive Interview -25 Items, *HVLT-R* Hopkins Verbal Learning Test—Revised, *HRR* Heart rate reserve, *IG* Intervention group, *LLT* Location Learning Test, *MMSE* Mini-Mental State Examination, *NI *No information, *RS* Rule Shift Card Test, *SCWT* Stroop Color and Word Test, *SDMT* Symbol Digit Modalities Test, *ST3* Stroop Test – third card, *TMT-A* Trail Making Test – Subtest A, *TMT-B *Trail Making Test – Subtest B, *VF* Verbal Fluency Test, VO_2_max Maximal aerobic capacity, *WAIS-III DST* Wechsler Adult Intelligence Scale – Third Edition Digit Span Test, *WAIS-R DST *Wechsler Adult Intelligence Scale – Revised Digit Symbol Test, WMS-R Wechsler Memory Scale—Revised Logical Memory, *WMS-III SS* Wechsler Memory Scale – Third Edition Spatial Span

^a^ Group differences in the changes marked as significant in publication

^b^ Group-based training was offered 2 times per week, but participants were encouraged to perform one session at home

### Effects of exercise on activities of daily living (ADLs)

The effects of moderate-to-high intensity exercise trainings over 16 weeks on ADLs were investigated in two studies [37, 42] (Table 3). Although Lamb et al. [42] found an improvement in physical fitness, these effects could not be translated into improvements in ADLs measured by Alzheimer’s Disease Cooperative Study ADL Scale (ADCS-ADL). These findings are in line with Hoffmann et al., who found no improvement in ADLs assessed with the Bristol Activities of Daily Living Scale (BADLS) [34].

**Table 3:** Included studies evaluating the effect of exercise programs on ADLs

Study	Type of training	Trial duration [weeks	Frequency [sessions/week]	Intensity	Session duration [min]	Setting	Adherence	Adherence	Outcome Baseline (mean ± SD)		Outcome follow-up (mean ± SD)		*P*-value	Conclusion
									IG	CG	IG	CG		
Hoffmann et al. [37]	Aerobic training	16	3	70–80% HRmax	60	Supervised groups of 2–5	76%	ADCS-ADL	64.8 ± 8.8	62.4 ± 10.8	64.4 ± 9.4	62.7 ± 10.4	.868	No effect
Lamb et al. [42]	Multimodal training	16	2 (+ 1)^a^	Moderate to hard	60–90	Supervised groups of 6–8 and unsupervised home-based	65.63%	BADLS	10	11	14.6 ± 9.5	14.6 ± 10.4	.15	No effect

*ADCS-ADL* Alzheimer’s Disease Cooperative Study ADL Scale,* BADLS* Bristol ADLs Scale, *CG* Control group, *HRmax* Maximal heart rate, *IG* Intervention group, *NI* No information

^a^ Group-based training was offered 2 times per week, but participants were encouraged to perform one session at home

### Effects of exercise on neuropsychiatric symptoms (NPS)

Four studies investigated the effects of aerobic training [37], multimodal training [42, 44], and chair-based strengthening or yoga exercises [38] on NPS, which were assessed by Neuropsychiatric Inventory (NPI) in three trials [37, 42, 44], while Park et al. [38] measured agitation, depression, and anxiety by Cohen-Mansfield Agitation Inventory-Short Form (CMAI) and Hospital Anxiety and Depression Scale (HADS), respectively (Table 4). Whereas Hoffmann et al. [37] described significant differences in change in total NPS, indicating less severe NPS in the intervention group after sixteen weeks of training, neither of the other interventions led to improvements in NPS after twelve [38], 16 [42], or 52 [44] weeks.

**Table 4:** Included studies evaluating the effect of exercise programs on NPS

Study	Type of training	Trial duration [weeks]	Frequency [sessions/week]	Intensity	Session duration [min]	Setting	Adherence	Assessment tool	Outcome Baseline (mean ± SD)		Outcome follow-up (mean ± SD)		*P*-value	Conclusion
									IG	CG	IG	CG		
Hoffmann et al. [37]	Aerobic training	16	3	70–80% HRmax	60	Supervised groups of 2–5	76%	NPI-12	10	9.4	8.8	11.4	.002^b^	Favors intervention
Lamb et al. [42]	Multimodal training	16	2 (+ 1)^a^	Moderate to hard	60–90	Supervised groups of 6–8 and unsupervised home-based	65.63%	NPI	8	10	12	8.5	.14	No effect
Öhman et al. [44]	Multi-modal training	52	2	NI	60	Supervised home based	72.88%	NPI	13.5 ± 12.6	16.6 ± 15.2	2.73 (1.08 to 5.05)^2^	0.64 (− 2.23 to 3.46)^c^	.41	No effect
						Supervised groups of 10	72.12%		12.1 ± 9.8		0.88 (− 1.30 to 2.84)^2^			
Park et al. [38]	Strength training	12	2	NI	45	Supervised groups	77.92%	CMAI	39.55 ± 8	42.89 ± 11.79	45.33 ± 11.96	46.4 ± 12.38	.09	No effect
									44.3 ± 8.74		53.25 ± 16.27		.47	
								HADS depression	6.82 ± 3.6	9.4 ± 4.09	8.33 ± 5	12.3 ± 4.76	.81	
	Chair yoga						76.25%		6.5 ± 2.51		8.38 ± 4.07		.89	
								HADS anxiety	4.36 ± 2.98	5.4 ± 2.63	6.78 ± 3.42	7.89 ± 3.18	.34	
									6 ± 4.27		8.5 ± 6.4		.12	

*CG* Control group, *CMAI* Cohen-Mansfield Agitation Inventory-Short Form, *HADS* Hospital Anxiety and Depression Scale, *HRmax* Maximal heart rate, *NPI* Neuropsychiatric Inventory, *NPI-12* Neuropsychiatric Inventory – 12 item version, *IG* Intervention group, *NI* No information

^a^ Group-based training was offered 2 times per week, but participants were encouraged to perform one session at home

^b^ Group differences in the changes marked as significant in publication

^c^ Change from baseline

### Risk of bias in included studies

Risk of bias varied between some to high concerns in the included studies (Table 5). For the detailed description, please refer to Additional file 1. Incompleteness of outcome data, selective reporting as well as measurement of the outcome were the predominant reasons for high concerns. Concerns in the measurement of the outcome mainly occurred because outcome assessors were aware of the intervention received. As participants were also aware of the assigned intervention, the possibility of bias due to deviations from the intended intervention did lead to some concerns in all included trials. Reporting of the trial of Karssemeijer et al. [39] and Lamb et al. [42] did not raise further concerns apart from blinding and thus should be considered as the included studies with the lowest risk of bias.

**Table 5:** Risk of bias assessment of the included studies

Study	Domain 1: Risk of bias arising from the randomization process	Domain 2: Risk of bias due to deviations from the intended intervention	Domain 3: Risk of bias due to missing outcome data	Domain 4: Risk of bias in measurement of the outcome	Domain 5: Risk of bias in selection of the reported result	Overall risk of bias
De Oliveira et al. [26]	Low concerns	High concerns	High concerns	High concerns	Some concerns	**High concerns**
Hoffman et al. [37]	Some concerns	Some concerns	Low concerns	Low concerns	Low concerns	**Some concerns**
Karssemeijer et al. [39]	Low concerns	Some concerns	Low concerns	Low concerns	Low concerns	**Some concerns**
Lamb et al. [42]	Low concerns	Some concerns	Low concerns	Low concerns	Low concerns	**Some concerns**
Öhman et al. [43, 44]	Low concerns	Some concerns	Low concerns	High concerns	High concerns	**High concerns**
Park et al. [38]	Some concerns	Some concerns	High concerns	High concerns	Some concerns	**High concerns**
Yu et al. [40]	Low concerns	Low concerns	High concerns	Low concerns	High concerns	**High concerns**

## Discussion

### Effects on cognition

This systematic review aimed to gather the current state of research on the effects of exercise on cognition, ADLs, and NPS in community-dwelling pwd. We found that pwd receiving exercise interventions did not yield additional benefits on global cognition in any of the included trials. In line with previous reviews, exercise could thus not be described as being effective for slowing down cognitive decline in pwd [12, 45]. Moreover, the results of Lamb et al. (2018) are of high interest, as they described a worsening of cognition in the intervention group after long-term follow-up and thus do not justify recommendation of physical exercise interventions as a treatment for cognitive decline in community-dwelling pwd.

Although our findings are consistent with Forbes et al. [12], they contrast with other studies and reviews [46, 47]. Differences in the study population might explain why our findings are inconsistent with other systematic reviews, which describe exercise as an effective treatment for cognitive decline in pwd without a formal diagnosis [46, 47]. De Oliveira et al. [41] did find significant improvements in cognition after a multimodal training intervention in people with MCI, but not in those with dementia and thus concluded that physical exercise should only be recommended in the early stages of neurocognitive disorders. Therefore, it seems useful to distinguish between people with MCI and pwd, since people affected experience different symptoms and biological adaptions so that possible mediators by which physical activity improves cognition may occur differently as the disease progresses [48]. In order to establish clear evidence and recommendations for physical activity in pwd, it is necessary to analyze a homogenous group in terms of diagnosis, as trainings recommendations might not be applicable for pwd as for people with MCI [49].

However, occasional significant superiorities for intervention groups in cognitive subdomains could be identified in two trials [39, 43]. Analyzing cognition, three trials used supervised sessions in groups [37, 40, 42], one did not report how the intervention was delivered [41], one compared two different settings [43], and one performed exercise individually guided [39]. Apart from Öhman et al. [43], none of the included trials reported beneficial effects for executive functions. These inconsistencies could have been arisen through the trainings content or the duration of the trial, as this was the only trial including training of executive function as part of the intervention, which lasted 52 weeks. Nevertheless, it needs to be stated that this multimodal training did lead to improvements in executive function for the home-based intervention group, while the same intervention did not affect executive function in a group-based setting. Deriving from this, there is an indication that training intervention for community-dwelling pwd are most beneficial if they are delivered individually guided and customized through a healthcare provider or the person’s caregiver. This hypothesis may further explain deviating findings from Karssemeijer et al. [39], who described, in contrast to other trials [37, 40], a significant amelioration in psychomotor speed after 12 weeks of aerobic training in the intervention group. Since training modalities, adherence to the protocol, design of control groups, measurements of the outcome, and exercises did not differ widely between the three trials, individual training sessions seem to be preferable as they might not overwhelm pwd and allow individually adapted designs.

### Effects on activities of daily living

In contrast to a previous Cochrane review [12], we could not find any beneficial effects on ADLs through physical activity in pwd. According to this review, there has been an unexplainable high heterogeneity between included studies, with only two trials [50, 51] out of six [52–55] favoring exercise over control. Furthermore, the sample size of studies included in the Cochrane review was comparatively small, ranging from six to 56 participants in the intervention group. Especially the power of the two studies observing beneficial effects on ADLs is limited, due to the sample size of eight [50] and eleven [51] participants in the intervention group. Therefore, Forbes et al. [12] suggested that these findings should be interpreted with caution and rated the quality of the evidence as low. Despite larger sample sizes in the included trials of this this review, ranging from 107 [37] to 329 [42], there were only two trials analyzing the effects of exercise on ADLs in pwd. We could not find an impact of adherence to the protocol.

### Effects on neuropsychiatric symptoms

Trials investigating the effects of exercise on NPS showed inconsistent results. Since training modalities such as duration, intensity, and setting did not differ widely between trials, different types of training might explain divergences. While strength and multimodal trainings intervention showed no beneficial effects on NPS [38, 42, 44], Hoffmann et al. [37] described a reduction of NPS after 16 weeks of aerobic training. Since only one trial analyzed the impact of aerobic exercise on BSPD, it seems possible that aerobic exercise might be effective to reduce NPS in pwd and this is in line with a recently published review [56]. Even though this might seem plausible, we could not find evidence for differences between studies with active or passive control groups or deviations in exercise adherence.

### Limitations

Although the search was conducted in eight different databases and 7651 trials were identified, we cannot rule out the possibility that we might have missed relevant trials due to limitations in language and year of publication. This might also be applicable for the requirement of a formal diagnosis of dementia. Some trials were excluded in this review because they included participants based on the results of screening instruments.

## Conclusion

### Implications for practice

There is little evidence that both strength and aerobic exercise or a combination of these cannot be recommended as a treatment option for cognitive impairment in community-dwelling pwd. Furthermore, moderate to high-intensity interventions might even worsen the cognitive decline in community-dwelling pwd after finishing the intervention [42]. In this context, it is mentionable that this was the study with the highest methodological quality and largest sample size. Furthermore, there is no evidence for the beneficial effects of exercise for ADLs. The effects on NPS are unclear, as one out of three studies found improvements after aerobic training. That is why healthcare providers and caregivers should be confident to promote the maintenance of an active and healthy lifestyle [57] among community-dwelling pwd instead, although recent recommendations [58] of moderate-intensity aerobic exercise for community-dwelling pwd are not underpinned by the results of this review. The development of best practice guidelines for healthcare providers is urgently needed. Exercise adherence does not seem to influence these outcomes.

### Implications for research

As our review shows, there is a necessity for improvement in methodological approaches in the research of the effects of exercise on cognition, ADLs, and NPS in community-dwelling pwd. Due to its large sample size and high methodological quality, the trial of Lamb et al. [42] should be considered as a best practice example. Recent research recommends at least 150 min/week of moderate-intensity aerobic exercise for older people, but this might not be appropriate for community-dwelling pwd [58]. Following on from this, future RCTs should require a formal diagnosis of dementia and should distinguish between pwd and people with MCI, as the conditions lead to different capabilities and needs, so that effects of exercise could therefore result in different outcomes [41]. High methodological quality, large sample sizes and long-term follow-ups should be implemented in future trials. In respect to possible impacts of social stimulation and activities on cognition and NPS, control groups should be designed accordingly to the intervention group. Especially if supervised group sessions are analyzed in a trial, control group should receive comparable social stimulation. To give answer to the question which type of training is most beneficial for community-dwelling pwd, it would be necessary that training modalities are described in detail and to compare different exercise protocols within three-armed RCTs. To compare different exercise programs and to be able to transfer research results in practice, it is inescapable to give a detailed description of the content and exercises of the trials, as it was the case in most of the included studies.

### Registration

This review was registered at the national prospective register of systematic reviews and no amendments were made (PROSPERO registration number: CRD42021246598). To view please visit https://www.crd.york.ac.uk/prospero/display_record.php?RecordID=246598.

No protocol was published in advance.

## Supplementary Information

**Additional file 1.** Risk of bias assessment. This additional file provides the detailed description of risk of bias assessment after discussion of both authors (K.S. and N.D.).

**Additional file 2.** Search strategy. This file provides the full search strategy for each database with detailed description (e.g. additional filters and dates of search).

## Data Availability

Data sharing is not applicable to this article as no datasets were generated or analyzed during the current study. This paper has not been previously published and is not currently under consideration for publication elsewhere.

## Abbreviations

ADAS-Cog: Alzheimer’s Disease Assessment Scale
ADCS-ADL: Alzheimer’s Disease Cooperative Study ADL Scale
ADLs: Activities of daily living
BADLS: Bristol Activities of Daily Living Scale
CMAI: Cohen-Mansfield Agitation Inventory-Short Form
HADS: Hospital Anxiety and Depression Scale
MCI: Mild cognitive impairment
MMSE: Mini-Mental Status Examination
NPI: Neuropsychiatric Inventory
NPS: Neuropsychiatric Symptoms
PRISMA: Preferred Reporting Items for Systematic Reviews and Meta-Analysis
PROSPERO: National Prospective Register of Systematic Reviews
pwd: People with dementia
RCTs: Randomized controlled trials
RoB2: Revised Cochrane Risk-of-Bias Tool for Randomized Trials
SCI: Subjective cognitive impairment
